# Wolfram syndrome 1: from ER stress to impaired mitochondrial dynamics and neuronal development

**DOI:** 10.1186/2193-1801-4-S1-P22

**Published:** 2015-06-12

**Authors:** Mailis Liiv, Michal Cagalinec, Zuzana Hodurova, Annika Vaarmann, Merle Mandel, Akbar Zeb, Malle Kuum, Miriam Ann Hickey, Dzhamilja Safiulina, Vinay Choubey, Eero Vasar, Vladimir Veksler, Allen Kaasik

**Affiliations:** Department of Pharmacology, Centre of Excellence for Translational Medicine, Institute of Biomedicine and Translational Medicine, University of Tartu, Estonia

**Keywords:** mitochondrial dynamics, ER stress, neuronal development

Wolfram syndrome 1 (WFS1) is a genetic disorder which has been associated both with impaired early brain development and neurodegeneration. Recent studies have suggested regulation of Ca2+ homeostasis by wolframin (Wfs1) and have demonstrated the involvement of endoplasmatic reticulum (ER) stress in Wfs1 deficiency. Despite the ER dysfunction WFS1 shows several characteristics of pathologies related to mitochondrial dynamics. Therefore our aim was to examine the hypothesis that Wfs1 deficiency could disturb mitochondrial dynamics contributing to impaired neuronal functioning. First we show that Wfs1 deficiency induces mild ER stress leading to Inositol 1,4,5-Trisphosphate Receptor (IP3R) dysfunction and disturbed cytosolic Ca2+ homeostasis, which, in turn alters mitochondrial trafficking, inhibits mitochondrial fusion and augments mitophagy. The overexpression of the active IP3R fragment restores IP3R-mediated Ca2+ release and corrects all perturbations in mitochondrial dynamics suggesting that these events are causally linked. We further demonstrate that suppressing the expression of two Parkinson disease-related proteins, Pink1 and Parkin, leads to reduced Wfs1 deficiency-induced mitophagy and also to the correction of the fusion-fission dynamics and mitochondrial motility. These data suggest that Wfs1 deficiency may over-activate Pink1 and Parkin pathways. Our most important discovery is that Wfs1 deficiency delays neuronal development and axonal growth in primary rat cortical neurons. According to our data, the link between Wfs1 deficiency and delayed neuronal development appears to be mediated by impaired mitochondrial dynamics because suppression of the Pink1-Parkin pathway corrected also the developmental delay. Our data shed light on the mechanisms of neuronal abnormalities in WFS1 and point out potential therapeutic targets. This work may have broader implications for understanding the role of mitochondrial dynamics in neuropsychiatric diseases.

